# Lidocaine for systemic sclerosis: a double-blind randomized clinical trial

**DOI:** 10.1186/1750-1172-6-5

**Published:** 2011-02-07

**Authors:** Rachel Riera, Luís EC Andrade, Alexandre WS Souza, Cristiane Kayser, Edison T Yanagita, Virgínia FM Trevisani

**Affiliations:** 1 Rheumatologist, Research Assistant of The Brazilian Cochrane Center and Medical Doctor of Discipline of Emergency Medicine and Evidence-Based Medicine at Universidade Federal de São Paulo - Escola Paulista de Medicina (UNIFESP-EPM). São Paulo, Brazil; 2 Rheumatologist, Professor of Discipline of Rheumatology at Universidade Federal de São Paulo - Escola Paulista de Medicina (UNIFESP-EPM). São Paulo, Brazil; 3 Rheumatologist, Medical Doctor of Discipline of Rheumatology at Universidade Federal de São Paulo - Escola Paulista de Medicina (UNIFESP-EPM). São Paulo, Brazil; 4 Rheumatologist, Medical Doctor of Discipline of Rheumatology at Universidade Federal de São Paulo - Escola Paulista de Medicina (UNIFESP-EPM). São Paulo, Brazil; 5 Medical Doctor at Universidade Federal de São Paulo - Escola Paulista de Medicina (UNIFESP-EPM). São Paulo, Brazil; 6 Rheumatologist. Full professor and Head of Discipline of Rheumatology at Universidade de Santo Amaro. Medical Doctor of Discipline of Emergency Medicine and Evidence-Based Medicine at Universidade Federal de São Paulo - Escola Paulista de Medicina (UNIFESP-EPM). São Paulo, Brazil

## Abstract

**Background:**

Systemic sclerosis (scleroderma; SSc) is an orphan disease with the highest case-specific mortality of any connective-tissue disease. Excessive collagen deposit in affected tissues is a key for the disease's pathogenesis and comprises most of the clinical manifestations. Lidocaine seems to be an alternative treatment for scleroderma considering that: a) the patient's having excessive collagen deposits in tissues affected by scleroderma; b) the patient's demonstrating increased activity of the enzyme prolyl hydroxylase, an essential enzyme for the biosynthesis of collagen; and c) lidocaine's reducing the activity of prolyl hydroxylase. The aim of this study was to evaluate the efficacy and safety of lidocaine in treating scleroderma.

**Methods:**

A randomized double-blind clinical trial included 24 patients with scleroderma randomized to receive lidocaine or placebo intravenously in three cycles of ten days each, with a one-month interval between them. Outcomes: cutaneous (modified Rodnan skin score), oesophageal (manometry) and microvascular improvement (nailfold capillaroscopy); improvement in subjective self-assessment and in quality of life (HAQ).

**Results:**

There was no statistically significant difference between the groups for any outcome after the treatment and after 6-months follow-up. Improvement in modified Rodnan skin score occurred in 66.7% and 50% of placebo and lidocaine group, respectively (*p *= 0.408). Both groups showed an improvement in subjective self-assessment, with no difference between them.

**Conclusions:**

Despite the findings of a previous cohort study favouring the use of lidocaine, this study demonstrated that lidocaine at this dosage and means of administration showed a lack of efficacy for treating scleroderma despite the absence of significant adverse effects. However, further similar clinical trials are needed to evaluate the efficacy of lidocaine when administered in different dosages and by other means.

## Background

Systemic sclerosis (scleroderma; SSc) is an orphan disease with the highest case-specific mortality of any connective-tissue disease [1]. It is a generalised disorder affecting small arteries, microvessels and diffuse connective tissue and is characterised by fibrosis and vascular obliteration in the skin, gastrointestinal tract, lungs, heart and kidneys, in which hidebound skin is the most distinctive clinical characteristic and organ degeneration is the basis for prognosis [2]. Its aetiology is unknown, however excessive collagen deposit in affected tissues is a key for the disease's pathogenesis and comprises most of the clinical manifestations of this disease, determining its development and prognosis; concurrently, numerous treatment attempts target these collagen deposits during various stages of the disease [3].

Treatment is difficult, and previous studies have often failed to demonstrate improvements to the skin or other organs using a variety of therapies, including immunosuppressive agents, putative antifibrotic drugs and highly specific, targeted biological therapies [4].

A cohort study, published in 1977, demonstrated that lidocaine appears to be an alternative treatment for patients with scleroderma - associated with an improvement in the cutaneous disorders and oesophageal manifestations and with no adverse effects [5]. This experimental cohort study included 15 patients with SSc and one patient with localized scleroderma. Participants received lidocaine 2% without adrenaline intravenously, diluted in 500ml of glucose solution (5% concentration) during six hours of infusion, for ten consecutive days, according to the following doses: 400mg on the first day, 480mg on the second, 560mg on the third, 640mg on the fourth, 720mg on the fifth and 800mg on the last five days. This cycle was repeated after 30, 60, 120 and 180 days and then every six months [5]. The outcomes were assessed before the treatment and after each cycle. According to the subjective evaluations made by the investigator and the patient independently, there was improvement in skin thickening in 75% of patients, in the oral cavity opening in 69% and in dysphagia in 66% of them. There was no change in the radiographic and electrocardiographic patterns and no occurrence of moderate or severe adverse effects during and after the infusions as well.

The rationale for this treatment presupposes three conditions: a) the patient's having excessive collagen deposits in tissues affected by scleroderma [3]; b) the patient's demonstrating increased activity of the enzyme prolyl hydroxylase, an essential enzyme for the biosynthesis of collagen, in the body' tissues [6]; and c) lidocaine's reducing the activity of prolyl hydroxylase [7].

Since the publication of this study, this method of intervention had been used by the Rheumatology Department of the Federal University of Sao Paulo as an alternative treatment for scleroderma patients with cutaneous and oesophageal manifestations.

Given the severity of the disease, its high mortality rate and the absence of effective treatments for scleroderma, it is relevant to assess the effectiveness of new therapeutic approaches. Therefore, the aim of this study was to evaluate the efficacy and the safety of lidocaine in treating scleroderma and thus increase the level of evidence for this intervention available in medical literature.

## Methods

A double-blind randomized clinical trial conducted in accordance with the Declaration of Helsinki and the CONSORT Statement (Consolidated Standards of Reporting Trials) [8], following approval by the Local Ethics Committee and trial registered in the Clinical Trials Database (NCT00740285).

### Patients

Eligible for participation were those patients with diffuse or limited SSc according to the American College of Rheumatology (ACR) criteria [9]. Inclusion criteria: age between 18 and 60 years old and time of onset of first sign or symptom less than five years. Exclusion criteria: SSc without cutaneous manifestations or caused by external agent; mental illness; pregnancy; overlap with other connective tissue disease; fibromyalgia; mandatory use of beta-blockers or cimetidine; or previous use of lidocaine, cyclophosphamide, d-penicillamine at any point over the previous six months. The use of the following concurrent treatment was not allowed during the study: methotrexate; azathioprine; chlorambucil; cyclosporine; interferon gamma; 5-fluorouracil; dimethyl sulfoxide; cyclofenil; n-acetylcysteine; para-aminobenzoate; vitamin D3; isotretinoin; anti-thymocytes globulin; cyclophosphamide; or d-penicillamine. Criteria for withdrawal from the study: the mandatory use of one of the aforementioned drugs during the trial.

### Interventions

After a full informed consent, participants were randomized in two groups: a) lidocaine 2% without vasoconstrictor; b) placebo. The interventions was administered intravenously for four hours, with 20mL administered during the first five days and 30mL during the last five days of treatment, in three cycles of ten days each, with a one-month interval between them. The drug and placebo were diluted in 500mL of a 0.9% saline solution.

### Randomization, Blinding and Allocation Concealment Procedures

To ensure homogeneity between the groups, randomization was matched and conducted by an independent researcher who did not know the patients. The following variables were used for pairing: patient's age; type of involvement (limited or diffuse); use of corticosteroids; and duration of the disease since its first manifestation or symptom.

After the randomization, the bottles were identified by the randomizer with the initials of the patient and they were delivered to the nurse in charge of the administration. The two interventions had the same physical appearance, colour and packaging. The patients, the nurse who administered the medications and the researchers who assessed the outcomes were blinded as to the intervention received by each patient.

### Outcomes

All outcomes were assessed before the intervention, immediately following the final treatment and after the 6-months follow-up period. The primary outcome was a decrease in the modified Rodnan skin score (RSS-m) by at least 30% immediately following three cycles of treatment. This score evaluates cutaneous thickening in 17 body areas, by clinical palpation, utilising a rating ranging from 0 (normal thickness) to 3 (extreme thickening) [10]. Consistent with the clinical trial guidelines, to avoid intra-observer variation and to minimise inter-observer variation, the score was applied twice to each patient blindly by a single researcher with eight months of training [11].

The secondary outcomes were: a) an increase of at least 30% in lower oesophageal sphincter pressure (at average airway pressure) and an increase of at least 30% in the average amplitude of peristaltic contractions. These outcomes were evaluated through an oesophageal manometry (Medtronic/Gastrosystem DS-8800 Plus, microcapillary infusion system- USA) carried out by a single researcher with ten years of experience in the method; b) a decrease of at least 30% in the number of enlarged capillary loops and capillary dropout, as assessed by nailfold capillaroscopy (stereomicroscope Olympus - SZ40 under 10-20x magnification) conducted by a single researcher, with five years of experience in the method; c) an improvement of quality of life of at least 30% on the Health Assessment Questionnaire (HAQ), which was administered by a single researcher after eight months of training; d) an improvement of subjective global self-assessment as reported in a self-administered questionnaire, which investigated patients' opinion about oral cavity opening, skin pigmentation and thickening, gastroesophageal complaints, arthralgia and the biggest drawback in the study; e) adverse effects as assessed through direct questioning of patients after each cycle and via reports from the attending nurse after spontaneous complaints during intervention.

### Statistical Analysis

To calculate the sample size, we estimated a frequency of 75% of the primary outcome in the lidocaine group and a frequency of 20% in the placebo group, as reported in the literature [5]. A type-1 error of 5% (alpha) and type-2 error of 10% (beta) were assumed - in other words, a significance level of 0.05 (p) and a sample power of 90%. Therefore, the sample was composed of 12 participants for each branch.

To assess the characteristics of data distribution (Gaussian or otherwise), the Kolmogorov-Smirnov and Shapiro-Wilk tests were applied.

The intra-class correlation coefficient was used to evaluate the homogeneity of the values of SCR-m obtained in both periods by the same investigator (intra-observer variation) and the Pearson correlation test as well. To allow this analysis, previously the Student T test was applied to compare the mean scores obtained between the two moments.

To compare the frequency of improvement in all outcomes between the groups (improvement of at least 30%), we used the Chi-square test or Fisher's exact test if necessary.

To compare the average results obtained between groups (named group effect) and also at the same time between two time points (immediately after intervention and after 6-months), the analysis of variance (ANOVA) with repeated measures was applied, considering that the measures over time were related to the same patient.

The Fisher's exact test was used to compare the frequency of adverse events between the two groups and also the subjective issues of the self-assessment questionnaire.

For all tests a significance level of 5% was considered and an intention-to-treat analysis was performed.

## Results

The study included 12 patients in each of the two groups, which were similar in terms of the main variables (p > 0.05) due to paired randomization and there were no withdrawals (Table 1).

**Table 1:** Baseline characteristics of patients

	*Placebo group (n = 12)*	*Lidocaine group (n = 12)*
Gender, n (%)		
Female	10 (83.4%)	10 (83.4%)
Male	2 (16.6%)	2 (16.6%)
Age, mean years ± SD	41.2 ± 11.1	40.8 ± 7.9
Type of involvement, n (%)		
Limited	4 (33.3%)	3 (25%)
Diffuse	8 (66.6%)	9 (75%)
Duration of the disease since manifestation of first symptoms, mean months (± SD	39.8 ± 15.9	41.5 ± 13.7
Use of corticosteroids, n (%)	8 (66.6%)	8 (66.6%)
Modified Rodnan skin score, mean ± SD	19.41	17.12
Capillary dropout, mean ± SD	1.8 ± 1.0	1.3 ± 1.1
Enlarged capillary loops, mean ± SD	4.04	2.90
Average amplitude of peristaltic contractions, mean mmHg (± SD	48.2 ± 38.8	47.6 ± 49.2
Lower esophageal sphincter pressure (at average airway pressure), mmHg mean ± SD	14.3 ± 6.9	13.6 ± 10.1
Health assessment questionnaire, mean ± SD	0.6 ± 0.4	0.8 ± 0.5

SD: standard deviation

### Modified Rodnan Skin Score (RSS-m)

All the applied tests indicated a high agreement between the two measures performed on each moment by the same researcher (Table 2). The Pearson correlation test between the two measures was 0,991 (before the treatment), 0,994 (immediately after the treatment) and 0,997 (after the 6-months follow-up period). The intra-class correlation coefficient was 0,991 (before the treatment), 0,994 (immediately after the treatment) and 0,999 (after the 6-months follow-up period).

**Table 2:** Intra-observer variation for modified Rodnan skin score

	*n*	*Mean*	*Median*	*SD*	*Lower Value*	*Higher Value*	P value *
**RSS- m 1a**	24	18,33	19	8,07	3	33	0,588
**RSS-m 1b**	24	18,21	17,5	8,17	3	33	
							
**RSS-m 2a**	24	13,29	13,5	6,4	2	30	0,257
**SCR-m 2b**	24	13,46	13,5	6,33	2	30	
							
**RSS-m 3a**	24	13,5	13,5	7,25	2	30	0,266
**RSS-m 3b**	24	13,38	13,5	7,29	2	30	

* T Student test

RSS-m 1a: modified Rodnan skin score - before treatment at moment 1; RSS-m 1b: modified Rodnan skin score - before treatment at moment 2; RSS-m 2a: modified Rodnan skin score - immediately after treatment at moment 1; RSS-m 2b: modified Rodnan skin score - immediately after treatment at moment 2; RSS-m 3a: modified Rodnan skin score - after 6-months follow-up at moment 1; RSS-m 3b: modified Rodnan skin score - after 6-months follow-up at moment 2; SD: standard deviation.

There was also no significant difference in the percentage of improvement by at least 30% in the RSS-m between the two groups (Table 3).

**Table 3:** Percentage of improvement in the RSS-m, oesophagus manometry and nailfold capillaroscopy considering evaluation at baseline and immediately following intervention

	*Placebo group, n %*	*Lidocaine group, n %*	*P-value*
**Improvement (decrease) in the RSS-m by at least 30%**			
**Yes**	8 (66.7%)	6 (50%)	0.408*
**No**	4 (33.3%)	6 (50%)	
**Improvement (increase) in lower oesophageal sphincter pressure by at least 30%**			
**Yes**	3 (25%)	6 (50%)	0.206*
**No**	9 (75%)	6 (50%)	
**Improvement (increase) in the amplitude of peristaltic waves by at least 30%**			
**Yes**	2 (16.7%)	4 (33.3%)	0.346*
**No**	10 (82.3%)	8 (66.7%)	
**Improvement (reduction) in the number of enlarged loops by at least 30%**			
**Yes**	2 (16.7%)	4 (33.3%)	0.346*
**No**	10 (83.3%)	8 (66.7%)	
**Improvement (reduction) in capillary avascular areas by at least 30%**			
**Yes**	2 (16.7%)	2 (16.7%)	1.00*
**No**	10 (83.3%)	10 (83.3%)	

* Fisher exact test

### Oesophageal Involvement

Regarding lower oesophageal sphincter (LES) pressure, there was no significant difference in the percentage of patients who showed an improvement (increase) of at least 30% in LES pressure between the two groups.

In relation to the average amplitude of the peristaltic waves, there was also no significant difference in the percentage of patients who showed an improvement (increase) of at least 30% in amplitude between the two groups. The values considered normal for this study's outcome ranged from 64 to 154mmHg (Table 3).

### Microvascular Changes

Each patient's fingernail beds were evaluated in order to obtain an average frequency of enlarged loops and capillary dropout. There was no significant difference in the percentage of patients who showed an improvement (reduction) by at least 30% in the number of enlarged loops and amount of capillary dropout between the two groups, when compared to the baseline data (Table 3).

### Quality of Life and Subjective Self-Assessment

No statistically significant difference was found in the percentage of patients who showed an improvement (decrease) of at least 30% in their HAQ score when compared to pre-intervention scores (p = 0.386).

The outcomes evaluated through the self-assessment questionnaire are presented in Table 4.

**Table 4:** Subjective self-assessment

	*Placebo group, n %*	*Lidocaine group, n %*	*P-value*
Improvement of oral cavity opening	6 (50%)	7 (58.3%)	0.682*
Improvement of skin pigmentation	8 (66.7%)	7 (58.3%)	0.673*
Improvement of skin thickening	12 (100%)	12 (100%)	1.00
Improvement of gastroesophageal complaints	5 (41.7%)	8 (66.7%)	0.219*
Improvement of joint pain	5 (41.7%)	7 (58.3%)	0.414*
The biggest drawback to trial participation			
None	4 (33.3%)	4 (33.3%)	1.00
Oesophageal manometry	8 (66.7%)	8 (66.7%)	
Would like to continue receiving treatment	12 (100%)	12 (100%)	1.00

* Chi-square test

### Adverse Effects

Adverse effects were rare (four patients in the lidocaine group and three in the placebo group), ephemeral and mild: anxiety; palpitations (no change in heart rate during the physical examination), itching; cramps; hypoesthesia; nausea; and dizziness (Table 5). Most effects occurred during treatment infusion, but there was no need to interrupt or postpone infusion.

**Table 5:** Frequency of adverse effects

		*Group*				
		*Placebo*		*Lidocaine*		*P value*
			
		n	%	n	%	
**Frequency of adverse effects**	No adverse effect	9	75,0%	6	50,0%	0,400 (F)
	At least one effect	3	25,0%	6	50,0%	

(F): Fisher exact test

Comparing the outcomes measures after the intervention and at 6-months follow-up (mid-term evaluation), no statistically significant difference between lidocaine and placebo groups were found and the values remained unchanged during the study period in both groups.

## Discussion

Despite the high morbidity and mortality associated with SSc and the lack of effective treatment for most manifestations of this disease, few adequately designed studies have been or are being conducted to address this shortcoming. A search of the Current Controlled Trials Database found only 17 related intervention studies, a similar search in Embase showed a mere 45 related trials, while The Cochrane Library found 242 trials from a database of more than 600,000 total trials (Table 6).

**Table 6:** Randomized clinical trials of scleroderma*

*Database*	*Search Strategy*	*Results*	RCT related*
**Cochrane Library**	scleroderma OR (systemic sclerosis) in Title, Abstract or Keywords not (multiple sclerosis) in Title, Abstract or Keywords in Cochrane Central Register of Controlled Trials (05/Sep/2010)	377	242
**Embase**	scleroderma OR (systemic sclerosis) Limits: Clinical Trial, Randomized Controlled Trial, Clinical Trial, Phase III, Clinical Trial, Phase IV, Controlled Clinical Trial (05/Sep/2010)	423	42
**Pubmed**	scleroderma OR (systemic sclerosis) Limits: Clinical Trial, Randomized Controlled Trial, Clinical Trial, Phase III, Clinical Trial, Phase IV, Controlled Clinical Trial (05/Sep/2010)	760	115
**Lilacs**	esclerodermia [Palavras] or (esclerose sistemica) [Palavras] and ( "ENSAIO CLINICO" ) or "ENSAIO CLINICO" or "ENSAIO CLINICO CONTROLADO" or "ENSAIO CLINICO CONTROLADO ALEATORIO" or "ENSAIO CLINICO FASE III" or "ENSAIO CLINICO FASE IV" [Tipo de publicação] (05/Sep/2010)	3	0
**Cilnical Trials Register Database**	scleroderma OR (systemic sclerosis) (05/Sep/2010)	140	104
**Current Controlled Trials Database**	scleroderma OR (systemic sclerosis) in ISRCTN Register (International) (05/Sep/2010)	38	17

RCT: Randomized clinical trials; Lilacs: Latin American and Caribbean Health Sciences Literature (Literatura Latino-Americana e do Caribe em Ciências da Saúde); * ECR really related with the theme - this was considered after the assessment of each abstract found and the full text, if necessary.

Foremost, the low prevalence of the disease and the delay in finding patients who meet the classification criteria make it difficult to include patients in studies of scleroderma. There was great difficulty in finding participants whose first symptom had manifest in fewer than two years prior to the study, two years being the timeframe recommended by the ACR [12]. However, we attempted to follow the recommendation for exclusion criteria exactly and to ensure that patients with characteristics that could lead to biased results were not included. We also followed the recommendations contained in the CONSORT Statement [8], and a stratified randomization was conducted in order to avoid heterogeneity between groups for characteristics that might influence results; all possible care was taken to maintain allocation confidentiality, and we are confident such was maintained; furthermore, an intention-to-treat analysis was conducted. The unpredictable course of the disease over time is undeniable regardless of the time of onset and thus we tried to become the groups as similar as possible to increase the internal validity without however reducing the external validity of results.

The lidocaine dosage and its means of administration were the same as those used in the previously published cohort study [5]. The number of cycles (three) was chosen by the researchers on account of the experience that this intervention had earlier shown that patients with no improvement in the first three cycles seemed to not respond subsequent cycles.

The outcomes were selected in accordance with the previously published cohort study, and the tools used for assessing these outcomes (RSS-m, nailfold capillaroscopy, oesophageal manometry and the HAQ) had already been validated [10,11,13-17]. Only one previously trained professional who had experience with the tool was indicated to assess each outcome. Furthermore, with respect to the RSS-m, the same observer twice assessed the same patient (in each assessment) and achieved good correlation pursuant to the Pearson test.

According to the Jadad scale, which assess the methodological quality of randomized controlled trials, this study achieved a score of 5, characterised by an adequate and explicit process of randomization, appropriate blinding and no losses [18]. Also, applying the risk of bias table proposed by the Cochrane Collaboration, this would be a low risk of bias clinical trial [19].

Regarding the results, which showed similar effects for the intervention and placebo groups through objective evaluation of outcomes, the authors do recognize the importance of the publication of randomized clinical trials with negative results, avoiding publication bias.

Also, it is important to highlight the following interesting observation. In the subjective self-evaluation and regardless of the intervention received, all patients reported improvement of skin thickening and, further, willingness to continue receiving the same treatment. Here we must stress the psychological side of the disease and ask whether the fact of having received more medical attention and more intensive care from health professionals might not have influenced patients' response to treatment. Intervention was conducted in a separate room in which a number of other scleroderma patients were receiving some type of treatment for other manifestations of the disease (e.g., cyclophosphamide for pulmonary involvement). Thus, treatment provided an opportunity to meet people with the same disease and with the same skin appearance, an aspect that elsewhere so often leads to stigmatisation. Feeling safer and less different, patients notoriously created bonds with others in similar situations and with the nurse in charge. We therefore believe that this aspect should be considered in this discussion.

Finally, as expected, considering the discomfort involved in administering treatment, patients reported that the study's biggest drawback was undergoing oesophageal manometry on three occasions.

Despite methodological care, we must point out some limitations in this study that should be avoided in future studies: a) During the sample size calculation was expected an improvement in primary outcome (RSS-m) of 75% in the intervention group (as reported in the literature) [5] and 20% in the placebo group (expected difference of 55%). However, the results showed a smaller difference (66.7% versus 50%, p = 0408), leading to a important reduction in the power of the sample initially calculated e used in this study; b) This trial included patients with more than two years since the first manifestation of the disease, contrary to the suggestion of the American ACR [12]; and c) the use of a fixed dosage of lidocaine, not one adjusted to each patient's weight.

Considering the findings of this clinical trial, our service centre has suspended the use of lidocaine as an alternative therapy for patients with cutaneous and oesophageal involvement in SSc.

## Conclusions

Despite the fact that the physiopathology, the researchers' initial experience (level of evidence V) [19] and the available cohort study (level of evidence IV) [20] favoured the use of medication, this clinical trial (level of evidence 1c) [20] has shown that lidocaine at this dosage and this means of administration was not effective in the treatment of skin, oesophageal and microvascular involvement in scleroderma or in improvement of patients' quality of life, despite the absence of significant adverse effects.

However, we believe further clinical trials, keeping the rigorous methodology, are needed to evaluate the effectiveness of lidocaine when administered in different dosages and by other means.

## List of Abbreviations

ANOVA: Analysis of variance; ACR: American College of Rheumatology; CONSORT: Consolidated Standards of Reporting Trials; (F): Fischer's exact test; FAPESP: State of Sao Paulo Research Support Foundation [Fundação de Apoio à Pesquisa do Estado de São Paulo]; HAQ: Health Assessment Questionnaire; LES: Lower oesophageal sphincter; RSS-m: Modified Rodnan skin score; SSc: Scleroderma, Systemic Sclerosis; SD: Standard Deviation; UNIFESP-EPM: Universidade Federal de São Paulo - Escola Paulista de Medicina

## Competing interests

There are no financial or non-financial competing interests (political, personal, religious, ideological, academic, intellectual, commercial or any other) related to this manuscript.

## Funding Sources

State of São Paulo Research Support Foundation [*Fundação de Apoio à Pesquisa do Estado de São Paulo*] (FAPESP), procedure number 01-13895-9

## Authors' contributions

RR has developed the design of the study, applied the RSS-m and the Health Assessment Questionnaire. Also performed the statistical analysis and prepared the paper.

LECA has made substantial contributions to revising the paper critically for important intellectual content.

AWSS has made substantial contributions to patient's selection and analysis of data.

CK has made substantial contributions to acquisition of data, performing the capillaroscopy.

ETY has made substantial contributions to acquisition of data, performing the manometry.

VFMT has made substantial contributions to conception and design and to paper development and has given final approval of the version to be published.

All authors read and approved the final manuscript.
